# Incorporating the Breast Imaging Reporting and Data System Lexicon with a Fully Convolutional Network for Malignancy Detection on Breast Ultrasound

**DOI:** 10.3390/diagnostics12010066

**Published:** 2021-12-28

**Authors:** Yung-Hsien Hsieh, Fang-Rong Hsu, Seng-Tong Dai, Hsin-Ya Huang, Dar-Ren Chen, Wei-Chung Shia

**Affiliations:** 1 Department of Information Engineering and Computer Science, Feng Chia University, Taichung 40724, Taiwan; p0100077@o365.fcu.edu.tw (Y.-H.H.); frhsu@fcu.edu.tw (F.-R.H.); m0806038@o365.fcu.edu.tw (S.-T.D.); 2 Comprehensive Breast Cancer Center, Changhua Christian Hospital, Changhua 500, Taiwan; 102982@cch.org.tw; 3 School of Medicine, Chung Shan Medical University, Taichung 40201, Taiwan; 4 Molecular Medicine Laboratory, Department of Research, Changhua Christian Hospital, Changhua 500, Taiwan

**Keywords:** breast cancer, deep convolutional network, image classification, semantic segmentation, ultrasonic imaging

## Abstract

In this study, we applied semantic segmentation using a fully convolutional deep learning network to identify characteristics of the Breast Imaging Reporting and Data System (BI-RADS) lexicon from breast ultrasound images to facilitate clinical malignancy tumor classification. Among 378 images (204 benign and 174 malignant images) from 189 patients (102 benign breast tumor patients and 87 malignant patients), we identified seven malignant characteristics related to the BI-RADS lexicon in breast ultrasound. The mean accuracy and mean IU of the semantic segmentation were 32.82% and 28.88, respectively. The weighted intersection over union was 85.35%, and the area under the curve was 89.47%, showing better performance than similar semantic segmentation networks, SegNet and U-Net, in the same dataset. Our results suggest that the utilization of a deep learning network in combination with the BI-RADS lexicon can be an important supplemental tool when using ultrasound to diagnose breast malignancy.

## 1. Introduction

Breast ultrasound (US) imaging is an important and common examination for the clinical diagnosis of breast cancer. It is a non-radiation imaging method, well tolerated by patients that can be easily integrated into interventional procedures [1]. However, the accuracy of breast US diagnoses is limited and dependent upon the experience and technical ability of the operator. Differences between operators, especially divergent skill, knowledge, and understanding of various breast US techniques, can lead to observer variations in diagnosis. A reliable computer-aided diagnosis (CAD) program can assist radiologists with image interpretation and diagnosis by providing a second objective opinion [2].

Deep learning has undergone rapid development with various and deeper network architecture and currently plays an important role in medical imaging analysis and computer-aid diagnosis. The classification of US images usually relies on a physician’s subjective evaluation. Deep learning generates a standardized analysis with objective and consistent results, and it can discover significant, hidden, provide a powerful reference in the clinic, and decrease observer bias.

Previous related studies used image segmentation [3,4] or lesion texture [5,6] to generate a pattern or model for malignant classification. In addition, several studies incorporated established significant features of the whole image into a deep learning network for malignant or benign tumor classification [7,8,9,10,11]. While all these previous studies had a classification accuracy of over 85% and showed good preliminary performance, providing only the benign and/or malignant classification of an image is insufficient for clinical practice. It is also important to determine whether the imaging findings match the characteristics of standardized terminology in the Breast Imaging Reporting and Data System (BI-RADS) [12], as well as the location or region of each imaging finding. 

The BI-RADS provides standardized terms (a lexicon) to describe breast mass features and assessments in radiology and effectively distinguishes between benign and malignant masses [13]. For a long time, the determination of these characteristics relied on the visual work conducted by the radiologist, and thus, the accuracy of the results was highly dependent on the physician’s experience and subjective judgment. A large amount of visual work also adds an additional burden on a busy medical center. Consequently, the recent development of semantic segmentation [14] may provide an important solution to this issue. Semantic segmentation can now classify each pixel of the image, divide the object, and indicate the location according to each target or feature to clarify the meaning of the whole image. Therefore, semantic segmentation is the basis of image understanding [15], making the digital image meaningful and simplifying its analysis. Recently, several semantic segmentation algorithms have been proposed, including an image-processing-based method and deep convolutional neural networks [14]. The combination of semantic segmentation and the BI-RADS lexicon can be used as the basis for the semantic segmentation analysis of breast US images to identify malignant or benign image characteristics that aid in the establishment of a patient’s clinical diagnosis.

This study aimed to combine semantic segmentation and deep learning to detect malignant-related image features from breast USs. The prediction result was visualized to help physicians distinguish malignancy on breast US and improve the quality of diagnosis in clinical practice. We applied a semantic segmentation network to detect malignant features based on the BI-RADS malignant lexicon definition in breast US images by utilizing a fully convolutional network. 

## 2. Materials and Methods

### 2.1. Data Acquisition

This retrospective, cross-sectional study was approved by the Institutional Review Board (IRB) of Changhua Christian Hospital, Changhua, Taiwan (No. 181235). The requirement for informed consent was waived by the ethics committee because of the retrospective nature of the study. All experimental methods were supervised by the IRB and conducted in accordance with the relevant guidelines and the Declaration of Helsinki.

The patients’ ages ranged from 35 to 75 years, and the benign or malignant classifications were pathologically proven (either by fine needle cytology, core-needle biopsy, or open biopsy). The full treatment, histology, and radiology records of all enrolled patients were also collected. Breast US images were acquired via the GE Voluson 700 system (GE Healthcare, Zipf, Austria). For each participant, at least two different scan plane angles were obtained. Each acquired breast US image showed the full screen of the scan plane. Each image had a resolution of 960 × 720 pixels in the RGB mode.

**Figure 1:**
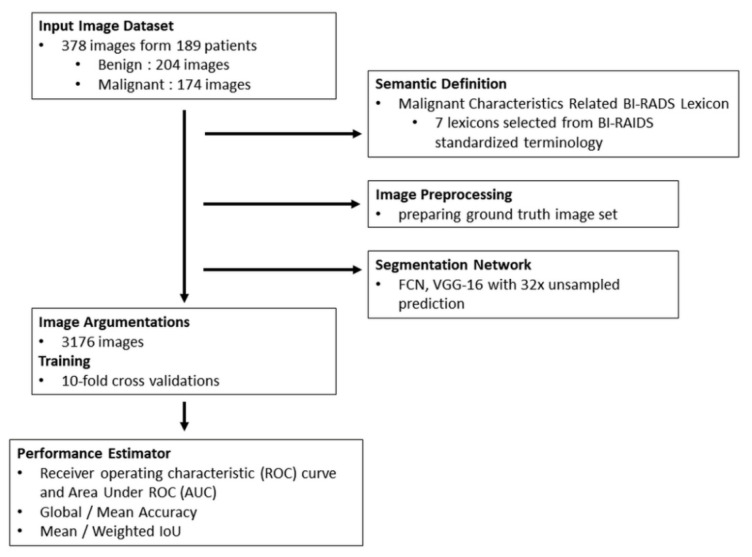
Study flowchart.

Malignant or benign cetology was classified according to the radiology and pathology report of each participant. All solid masses identified in US images were described by standardized terms, categorized according to the American College of Radiology (ACR) BI-RADS fifth edition category criteria [16,17], and verified by surgeons with over ten years of experience. The flowchart of the data process, analysis, and performance estimation is shown in Figure 1.

### 2.2. Definition of Semantics and Lexicons

The semantic definition was based on the BI-RADS lexicon of malignant characteristics in US, with a focus on the high-frequency lexicon present in radiology reports, which belongs to BI-RADS categories 4, 5, and 6. After the analysis, seven lexicons were selected from the following: shadowing, echogenic halo, taller-than-wide, non-parallel, circumscribed or indistinct tumor margin, angular margins, micro-lobulation, hypoechogenicity, and duct extension.

### 2.3. Data Pre-Processing and Argumentation

Non-related marks, such as the manufacturer mark, direction indicator, and text field, were cropped from the original image in pre-processing to prevent incorrect training. The final processed image used as input material was cropped to 560 × 560 pixels. The images did not include any pre-selected tumor region or label.

**Figure 2:**
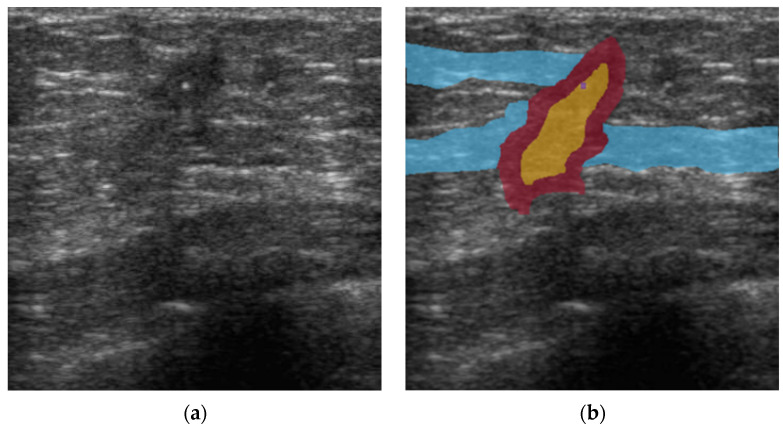
An example of an ultrasound image and ground truth image with corresponding Breast Imaging Reporting and Data System (BI-RADS) lexicon from a patient with malignant breast cancer. (**a**) sample image, (**b**) ground truth image, containing three BI-RADS lexicons: angular margin (red region), duct extension (light blue), and taller-than-wide (yellow).

The region of each malignant lexicon was manually sketched to correspond to the input US image and saved as the ground truth image. The source of malignant lexicons in each US image was based on the radiology report, and the correctness of the ground truth region and location was confirmed by an experienced radiologist. Figure 2 presents an example of a source US image and ground truth image from a patient with malignant breast cancer. The regions with corresponding BI-RADS lexicons were sketched in different colors according to the predefined color map. 

Due to the smaller dataset and increased segmentation performance during network training, we also applied image argumentation to the dataset before training, comprising random zooming (from 0.8× to 1.2×), rotation (−90 to 90 degrees), cropping, flipping (vertical and horizontal), and elastic distortion. After the image argumentation, the image dataset was increased to 3136 images. Then, 10-fold cross-validation was applied to the network training. All the required related programs in ground truth marking, image encoding, pre-processing, and argumentation was implemented in MATLAB 2019b update five with Image Processing Toolbox (The Math Works, Natick, MA, USA). 

### 2.4. Semantic Segmentation Networks

**Figure 3:**
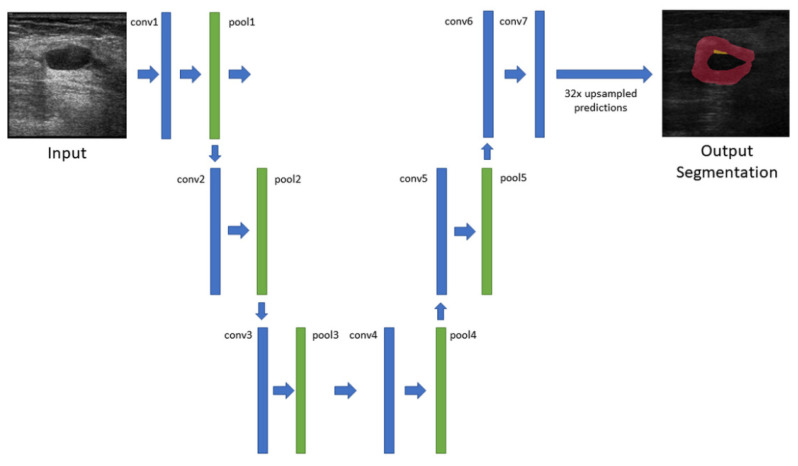
Network architecture of the fully convolutional network with 32× upsampled prediction.

The deep network utilized for the semantic segmentation in this study was a fully convolutional network (FCN) [18]. The architecture of FCN uses layers of VGG-16 [19] for convolutionalizing classification, with 32× upsampled prediction (FCN-32s). The image was reduced to a thirty second of the source after five pooling, and output upsampling was performed in the deconvolutional layer (conv 7) for end-to-end learning by backpropagation. The benefit of using an FCN for semantic segmentation is that it combines layers of the feature hierarchy and refines the spatial precision of the output, enabling the combination of coarse high-layer information with fine low-layer information by learning [18]. The network architecture is presented in Figure 3. All the required FCN architecture and related programs were implemented in MATLAB 2019b update five with the Deep Learning Toolbox (The Math Works, Natick, MA, USA).

### 2.5. Performance Estimation

The semantic segmentation performance was estimated according to the ground truth image dataset. The metrics used for the estimation included the global accuracy, mean accuracy, mean, frequency of the weighted intersection over union (IU), and mean boundary F1 score (BF score) [18,20]. These metrics were computed by utilizing related functions within the Computer Vision Toolbox and the Deep Learning Toolbox of MATLAB. The following equations were utilized, with n_ij_ as the number of pixels of class I predicted to belong to class j, when there are n_class_ different classes:

Global accuracy:

(Number of true classified pixel)/(Total number of pixel) = TP + TN/TP + TN + FP + FN(1)

Mean accuracy:

TP/TP + FN(2)

Mean IU:

Intersection/Union = 1/n_class_ ∑_I_ TP/TP + FP + FN (3)

Mean BF score:

(2 × Precision × (1 − Recall))/(Precision + (1 − Recall)) = 2TP/((TP + FN) + (TP + FP))(4)

where Precision = TP/TP + FP; Recall = TP/TP + FN; TP = True Positive; TN = True Negative; FP = False Positive; and FN = False Negative.

We also compared the diagnostic performance of the semantic segment networks and the ground truth by plotting the receiver operating characteristics (ROC). The criteria for correction were based on the frequency weighted IU. If the overlap region in the ground truth pixel region was >75% in each BI-RADS lexicon, the segmentation result was considered “correct,” while those with an overlap < 75% were considered “incorrect.”

### 2.6. Computation Environment

All computations were performed on an ASUS ProArt Studiobook Pro 15 laptop with an Intel Core i7-9750H processor (2.6 GHz hexa-core with up to 4.5 GHz Turbo Boost and 12 MB cache), 32 GB DDR4 ECC RAM, and NVIDIA Quadro RTX 5000 MAX Q graphic card with a 16 GB video RAM (Asus, Taipei, Taiwan). The NVIDIA Compute Unified Device Architecture (CUDA), version 10.2, and the NVIDIA CUDA Deep Neural Network library (CuDNN), version 10.2 enabled the accelerated computation environment of the graphics processing unit (GPU) (NVIDIA, San Jose, CA, USA).

## 3. Results

### 3.1. Characteristics of Image Set

In this study, after the exclusion criteria were applied to all the participants, the image dataset contained 378 images (204 benign and 174 malignant images) from 189 patients (102 patients with a benign breast tumor and 87 with a malignant one). In benign cases, the most common tissue types of solid nodes were fibroadenomas (28/102, 27.45%), fibrocystic changes (26/102, 25.49%), and fibroepithelial lesions (27/102, 26.47%). The incidence of lobular carcinoma in situ (LCIS) was 3.92% (4/102). In the case of malignant tissue types, the incidence of ductal carcinoma in situ (DCIS) was 20.82% (18/87); the most common of which was invasive ductal carcinoma (IDC) (69/87, 79.18%). Table 1 shows the detailed characteristics of the image dataset and patients. Table 2 shows the image amount, number of pixels, and total number of pixels in each lexicon. The most common malignant lexicons were angular margin and taller-than-wide.

### 3.2. Result

**Figure 4:**
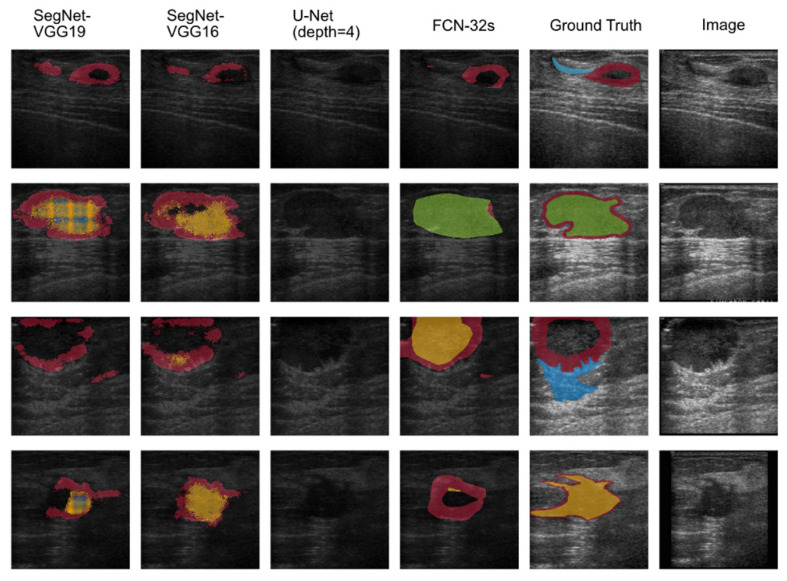
The visualization result of the semantic segmentation network output shows four original malignant tumor ultrasound images and the result after applying the semantic segmentation network. The original image and corresponded ground image are shown in the two columns on the far right. The semantic segmentation result from the first column on the left to the right: SegNet with VGG19 encoder, SegNet with VGG16 encoder, U-Net with depth equally four, and FCN-32s.

**Figure 5:**
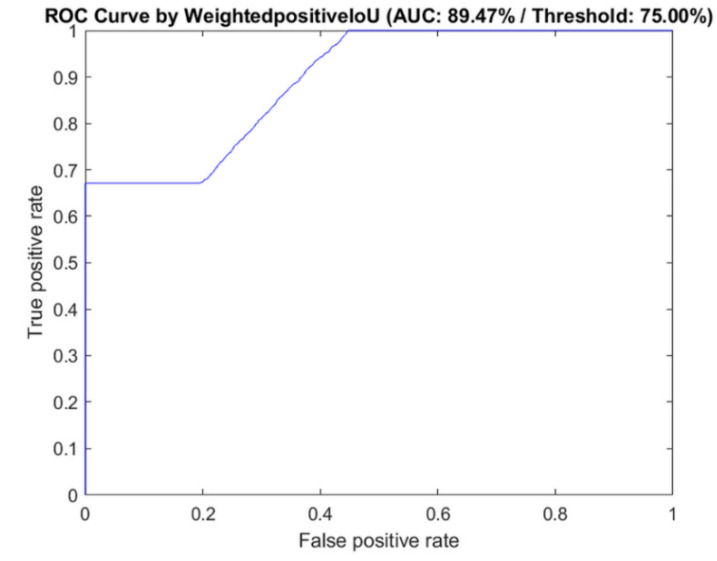
The ROC and AUC of the classification performance based on selected BI-RADS lexicons in the FCN. AUC: area under curve, SVM: support vector machine, BI-RADS—Breast Imaging Reporting and Data System, ROC: receiver operating characteristic curve.

The output of the semantic segmentation was also visualized by using a customized color map, which clearly displays the selected seven malignant features to help physicians judge the malignant tumor and corresponding BI-RADS category. Figure 4 presents the original US image and the semantic segmentation visualization result. Each lexicon has a different color filled in the detected region, according to the specific color map. The global accuracy of the FCN was 91.49%, the mean accuracy was 32.82%, and the weighted IU was 85.35%. The mean BF score was 61.02. Table 3 presents the performance of the segmentation networks evaluated in this study. The AUC of correct recognized BI-RADS lexicons was 89.47%. The sensitivity was 88.64%, and the specificity was 91.76% (*p* < 1 × 10^−5^). The PPV and NPV were 89.1% and 87.8%, respectively. The ROC curve and the AUC are shown in Figure 5.

### 3.3. Comparisons to Other Semantic Segmentation Networks

**Figure 6:**
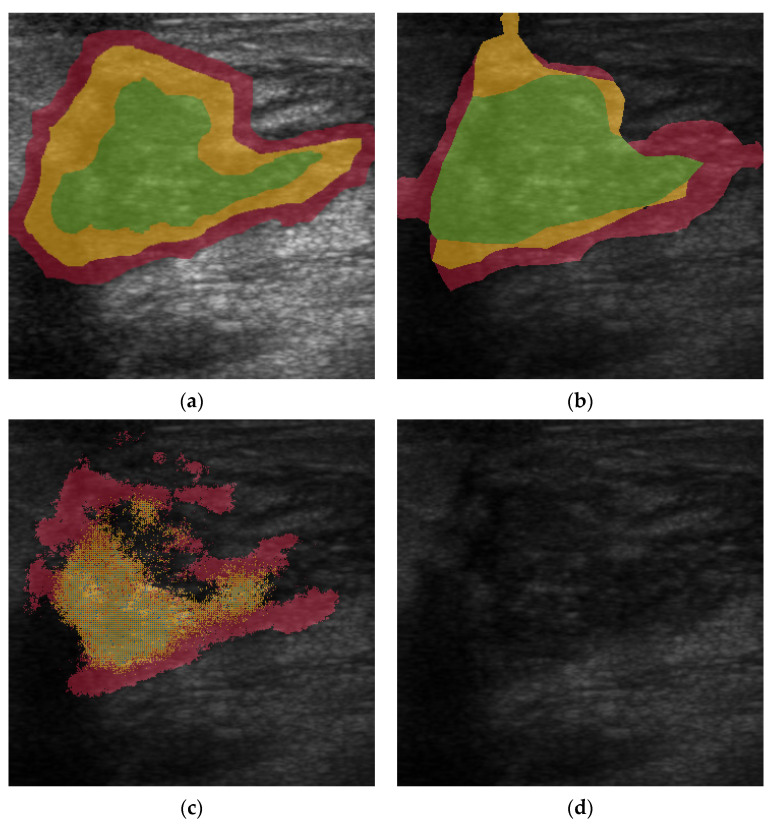
Comparison of segmentation network results in a specific patient with malignancy. (**a**) input ultrasound image and its ground truth. The input US image contains three BI-RADS lexicons: angular margin (in red), hypo-echogenicity (in green), and taller than wide (in yellow); (**b**) Segmentation by FCN-32s. All three characteristics of the BI-RADS lexicons were conserved. There is some deviation in the size of the region in each lexicon, and most of the segmentation was correct. (**c**) Segmentation by SegNet (based on VGG-19). Although the angular margin and taller-than-wide characteristics were recognized, there is no clear margin in each region of BI-RADS lexicon. Apart from the taller-than-wide region being broken, some of that region was also recognized as duct extension (in blue dots), which was not in the original input image. (**d**) Segmentation by U-Net (depth = 4). This network failed in this test.

Two recent semantic segmentation networks, SegNet [21] and U-Net [22], were also utilized to compare their performance to that of the FCN-32s used in this study. Figure 4 shows the segmentation network outputs of SegNet (based on VGG 16 and VGG19 encoder), U-Net (when depth = 4), and FCN-32s. Figure 6 illustrates the comparisons of the segmentation networks in a specific case (malignant patient #28). Overall, the FCN-32s showed good semantic segmentation performance and better detection of specific lexicons, such as angular margins and taller-than-wide, than SegNet or U-Net. Especially, it usually conserved complete and clear margins for each BI-RADS lexicon after image segmentation.

**Figure 7:**
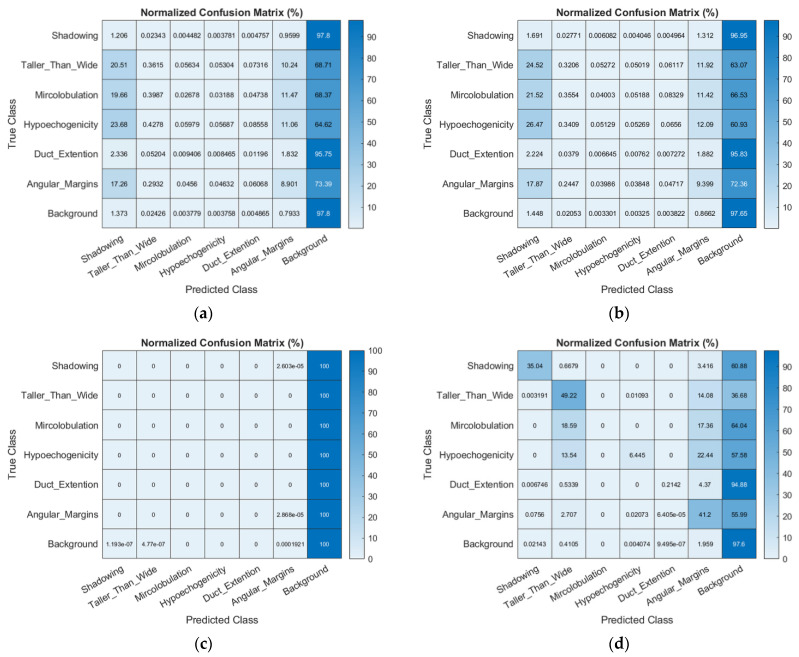
Normalized Confusion Matrix of the classification performance based on selected BI-RADS lexicons in SegNet, U-Net, and FCN-32s. The proportion of correctly recognized in each lexicon among the three networks (in percentage). (**a**) classification performance of SegNet with the VGG16 encoder; (**b**) classification performance of SegNet with the VGG16 encoder; (**c**) classification performance of U-Net; (**d**) classification performance of the FCN.

Figure 7 presents the normalized confusion matrices of all semantic segmentation networks in this study in order to illustrate the proportion of correct results in each recognized lexicon. The segmentation result utilizing SegNet with the VGG16 encoder only recognized angular margin and shadowing and showed low correctness (9.1% and 1.5%). Comparing the output to the ground truth result revealed that the hypoechogenicity region was covered and mixed by other lexicons, and most of the duct extension region was recognized as angular margins. Only some pixels belonging to duct extension and taller-than-wide, which were near the tumor margin, were correctly recognized. The segmentation result using SegNet with the VGG19 encoder showed higher accuracy in recognizing the angular margin. Most of the taller-than-wide and angular margin regions were incorrectly recognized as duct extension. This network presented good performance in feature segmentation (weighted IU: 79.54%, mean BF score: 80.77%); however, because the background comprised the largest region in each image, the normalized confusion matrix of the U-Net classified most of the feature pixels as background. This is an incorrect finding.

## 4. Discussion

In this study, we focused on the ability of semantic segmentation, combining deep network and the BI-RADS lexicon, to facilitate multi-target segmentation of US images by comparing the similarity of this prediction result to that of the radiology report drafted by experienced physicians. We also sought to provide a visualization of the detected malignant features by region for preliminary diagnostic reference. This visualization is clinically impactful, particularly for physicians and radiologists, because it can show all detected US image features that are synonymous with the BI-RADS malignant lexicon at a glance; this considerably decreases the effort of visually reading the image. In comparison, traditional image segmentation usually partitions, clusters, and locates objects on images by using segmentation methods (such as color, texture, and boundary smoothness) and does not tag the region or fragment that belongs to the same or related cluster, thus lacking in-depth meaning for these segmentation regions. Furthermore, when malignant characteristics are detected and found to be synonymous with specific BI-RADS lexicons, traditional image segmentation cannot segment more than one feature from a single image.

Our preliminary result showed that semantic segmentation could segment multiple malignant image features from one image, and these malignant features were synonymous with specific BI-RADS terms. The global accuracy, weighted IU, and AUC of the FCN-32s were over 85%, showing an acceptable performance, which was better than that of SegNet and U-Net after the estimations. The mean accuracy and mean IU of the FCN-32s in this study was slightly less than the average level found in a related study [23] (32.82% vs. about 40% and 28.88 vs. approximately 35, respectively) due to an imbalance in the image count and pixel count in each lexicon. The most frequently cited characteristic was angular margin (366/378, 96.82%), and the least frequent were shadowing (14/378, 3.7%) and microlobulation (17/378, 4.5%). In this situation, using the mean accuracy and mean IU as performance metrics led to inaccurate estimates; the use of the weighted IU was more appropriate.

In cases with a malignant breast mass, it is common to have both malignant and benign image features in the same report [12]. Therefore, classifying the malignant or benign tumor in the US image merely according to the detected BI-RADS malignant lexicon is inappropriate; the classification must be confirmed by pathology. The image dataset in this study included both benign and malignant tumors in similar proportions; thus, our results suggest that this segmentation procedure is suitable for both benign and malignant tumor images and meets the requirements for daily clinical use.

The main limitation of this study was its small image dataset and the partial utilization and recognition of BI-RADS lexicons. Each image may have multiple malignant lexicon characteristics that need to be tagged and sketched related to their region and location, which increased the training dataset preparation and limited the size of the image dataset. In addition, only the seven BI-RADS characteristics most related to malignant tumors were selected. It is important to extend the number of detectable characteristics. The similarity score or rating to the ground truth after segmentation was also not provided in this study. At present, the weighted IU and mean BF score reached acceptable levels in this study, and the application of a small amount of data to FCN32s did not have much impact on the result analysis. However, the application to a larger dataset would result in more accuracy. These defects should be addressed in future work based on this study.

## 5. Conclusions

In contrast to traditional image segmentation, semantic segmentation of medical images is a more advanced and complicated task. The inherent noise and speckle of US imaging create indistinct margins around the malignant feature and increase the difficulty of segmentation. Therefore, it remains challenging to obtain meaningful diagnostic information from semantic segmentation of US imaging. In this study, the combination of deep learning and a semantic segmentation network with a pre-defined BI-RADS malignant-related lexicon to analyze US images was used to extract specific features from US images that were synonymous with the BI-RADS malignant terminology. The application of this network could help physicians make a fast and accurate diagnosis of malignant breast tumors.

## Figures and Tables

**Table 1:** Patient and image characteristics.

Characteristics	Benign (*n* = 102)	Malignant (*n* = 87)
Age of patients (y)	45.17 (43.28–47.75)	55.63 (53.25–57.84)
BI-RADS category		
3	21 (20.59%)	3 (3.01%)
4A	73 (71.57%)	31 (35.62%)
4B	5 (4.90%)	17 (12.60%)
4C	2 (1.96%)	10 (11.78%)
5	1 (0.98%)	26 (29.04%)
Malignant tissues		
DCIS	-	18 (20.82%)
IDC	-	69 (79.18%)
Benign tumors		
LCIS	4 (3.92%)	-
Fibroadenoma	28 (27.45%)	-
Fibrocystic change	26 (25.49%)	-
Adenosis	3 (2.94%)	-
Fibroepithelial lesion	27 (26.47%)	-
Other	14 (13.73%)	-

BI-RADS: Breast Imaging Reporting and Data System; US: Ultrasound; DCIS: Ductal carcinoma in situ; LCIS: Lobular carcinoma in situ; IDC: Invasive ductal carcinoma.

**Table 2:** Selected lexicons in this study and the image/pixel count of the dataset.

Name	Pixel Count	Image Pixel Count	Image Count
Shadowing	184,157	1,814,400	14
Taller Than Wide	912,091	6,739,200	52
Microlobulation	7593	2,203,200	17
Hypo Echogenicity	153,534	4,406,400	34
Duct Extension	116,809	2,851,200	22
Angular Margins	3,706,365	47,433,600	366
Background	43,908,251	48,988,800	378

Pixel Count: Number of pixels in each lexicon. Image Pixel Count: The total number of pixels in each lexicon in all images. Image Count: Total number of images in each lexicon.

**Table 3:** Performance results in SegNet, U-Net, and FCN-32s.

Network	Global Accuracy	Mean Accuracy	Mean IU	Weighted IU	Mean BF Score
SEGNET (VGG16)	87.89%	15.48%	14.03	80.69	44.88
SEGNET (VGG19)	87.87%	15.65%	14.12	81.02	44.27
U-NET (DEPTH = 4)	89.18%	14.29%	12.74	79.54	80.77
FCN-32S	91.49%	32.82%	28.88	85.35	61.02

IU: intersection over union, BF score: boundary F1 score.

## Data Availability

The datasets generated during and analyzed during the current study are not publicly available due to IRB and institutional restrictions but are available from the corresponding author on reasonable request.
